# Brazilian Red Propolis Attenuates Hypertension and Renal Damage in 5/6 Renal Ablation Model

**DOI:** 10.1371/journal.pone.0116535

**Published:** 2015-01-21

**Authors:** Flávio Teles, Tarcilo Machado da Silva, Francisco Pessoa da Cruz Júnior, Vitor Hugo Honorato, Henrique de Oliveira Costa, Ana Paula Fernandes Barbosa, Sabrina Gomes de Oliveira, Zenaldo Porfírio, Alexandre Braga Libório, Raquel Lerner Borges, Camilla Fanelli

**Affiliations:** 1 Renal Division, Department of Clinical Medicine, Faculty of Medicine, State University of Health Sciences, Alagoas, Brazil; 2 Faculty of Medicine, Federal University, Ceará, Brazil; 3 School of Sport and Exercise Science, University of Northern Colorado, Greeley, United States of America; 4 Renal Division, Department of Clinical Medicine, Faculty of Medicine, University of São Paulo, São Paulo, Brazil; University of São Paulo School of Medicine, BRAZIL

## Abstract

The pathogenic role of inflammation and oxidative stress in chronic kidney disease (CKD) is well known. Anti-inflammatories and antioxidant drugs has demonstrated significant renoprotection in experimental nephropathies. Moreover, the inclusion of natural antioxidants derived from food and herbal extracts (such as polyphenols, curcumin and lycopene) as an adjuvant therapy for slowing CKD progression has been largely tested. Brazilian propolis is a honeybee product, whose anti-inflammatory, antimicrobial and antioxidant effects have been widely shown in models of sepsis, cancer, skin irritation and liver fibrosis. Furthermore, previous studies demonstrated that this compound promotes vasodilation and reduces hypertension. However, potential renoprotective effects of propolis in CKD have never been investigated. The aim of this study was to evaluate the effects of a subtype of Brazilian propolis, the Red Propolis (RP), in the 5/6 renal ablation model (Nx). Adult male Wistar rats underwent Nx and were divided into untreated (Nx) and RP-treated (Nx+RP) groups, after 30 days of surgery; when rats already exhibited marked hypertension and proteinuria. Animals were observed for 90 days from the surgery day, when Nx+RP group showed significant reduction of hypertension, proteinuria, serum creatinine retention, glomerulosclerosis, renal macrophage infiltration and oxidative stress, compared to age-matched untreated Nx rats, which worsened progressively over time. In conclusion, RP treatment attenuated hypertension and structural renal damage in Nx model. Reduction of renal inflammation and oxidative stress could be a plausible mechanism to explain this renoprotection.

## Introduction

Chronic Kidney Disease (CKD) is an important cause of death and disability worldwide. In the last years CKD reached epidemic proportions, at least partially due to population aging, obesity and rising incidence of diabetes and hypertension. According to the National Kidney Foundation, CKD meets all the required criteria to be considered as a major public health concern, which makes the elucidation of mechanisms involved in the progression of chronic nephropathies, as well as the development of new therapies to reduce its progression, urgently required [1,2].

The pathogenic role of inflammation and oxidative stress in CKD is well established. Both the administration of anti-inflammatory drugs and the pharmacological inhibition of ROS (reactive oxygen species) production have shown potential renoprotection in experimental models of nephropathy, including the 5/6 renal ablation model (Nx), in which the right kidney is surgically removed and the left kidney has two-thirds of it infracted by ligation of two or three branches of the left renal artery [3–9]. Nx is characterized by early development of hypertension, proteinuria, glomerulosclerosis, interstitial inflammation and fibrosis. However, according to Fujihara and collaborators early treatment of Nx rats with either Mycophenolate Mofetil (a potent immunosuppressor) or PDTC (a NF-kappaB inhibitor that also presents antioxidant effects) attenuates renal injury and slows the progression of CKD [5,6,10].

Brazilian propolis is a resinous mixture produced by *Apis mellifera* bees through the collection of variable plant sources [11–13]. It can be classified into 12 different types, according to physicochemical properties and the geographic location in which it is found. Propolis has been used in folk medicine for centuries and its anti-inflammatory properties, generally attributed to its large content of flavonoids and isoflavones, have been demonstrated in several experimental studies, including *in vivo* and *in vitro* models of sepsis, acid-induced colitis, stress-induced gastric mucosal lesions, salycilate-induced skin irritation and liver fibrosis [14–16]. This natural compound has also been reported to promote vasodilation and hemodynamic effects. As demonstrated by Kubota and, five years later, by Maruyama; administration of a Green Propolis-rich diet (0.5%) to Spontaneously Hypertensive Rats (SHR), during four weeks, reduced systemic blood pressure significantly [14,15].

Recently a new type of propolis, named Brazilian Red Propolis (RP) due to its color, was found in Maceio City (Alagoas state, Northeastern Brazil). This was considered the 13^th^ type of propolis, since it is made from a different botanical source (*Leguminosae family*). Its chemical composition was characterized by Silva and collaborators [17]. Ethanol extracts of RP was shown to present antibacterial and anti-parasitic properties; however, possible effects of RP on blood pressure and on the progression of renal disease, were not yet evaluated. Therefore, the present study aimed to investigate the effects of RP on the progression of nephropathy associated to the Nx model. Moreover, we aimed to clarify whether propolis administration would mitigate renal inflammation and thereafter the renal function loss on remnant kidney.

## Methods

### Animals and Surgical Procedure

Thirty-two adult male Wistar rats aged approximately 2 months and weighing 220–250g were provided by State University of Health Sciences of Alagoas to be included in this study. These animals were maintained at 22°C, under a 12/12-h light-dark cycle and had free access to potable water and standard rodent chow (0.5% Na, 22% protein) during all the experimental protocol. To obtain the Nx model, rats underwent a surgical procedure. The animals were first anesthetized with an intraperitoneal injection of Ketamin 5% (50 mg/kg) and Xylazin 2% (0.5 ml/kg), in the ratio of 8:1 and then subjected to a ventral laparotomy. The right kidney was removed and two-thirds of the left kidney was infarcted, by closing two or three branches of the left renal artery. Sham-operated rats (S: Used as control groups) underwent anesthesia and manipulation of the renal pedicles without renal mass reduction. All the experimental procedures performed in this study were approved by the local Research Ethics Committee (Institutional Animal Care and Use Committee of the State University of Health Sciences of Alagoas—UNCISAL- protocol number 54-A) and were developed in strict conformity with our institutional guidelines and with international standards for manipulation and care of laboratory animals. All rats were manipulated and weighted daily for monitoring the body weight gain and their general health condition. Rats in bad condition (reduced mobility, reduced food and fluid intake) and/or with the weight gain severely stunted (weight loss greater than 20% lasting more than a week after surgery) were euthanized by an overdose of anesthetic (intraperitoneal injection of Ketamin and Xylazin, in the ratio of 8:1, at 4 times the anesthetic dose).

### Red Propolis (RP) and Experimental Groups

Chemical composition of alcoholic RP extract employed in this study was: 20% of RP collected in a mangrove area of Alagoas, in northeastern Brazil; 27% distilled water and 53% ethanol. According to High Performance Liquid Chromatography (HPLC) analysis, the main constituents of RP extract are the isoflavonoids; medicarpin and 3-hidroxy-8, 9-dimethoxypterocarpan; the latter representing more than 60% of its composition [16]. The animals were distributed among the following groups: Sham (S, n = 8) and Nx (Nx, n = 11) untreated rats; Sham (S+RP, n = 8) and Nx (Nx+RP, n = 8) rats treated orally with 150 mg/kg/day of alcoholic RP extract diluted in drinking water. Water intake was measured daily to keep RP dosages constant. All groups were followed for a total period of three months.

Experimental studies employing propolis treatment in rodents are very variable regarding the dosage and route of administration of this compound [13–16]. The dose of 150 mg/kg/day was established on preliminary experiments as the maximum dose tolerated by animals without growth stunting or deterioration of their general condition. In order to establish this dose, we performed a pilot study before starting our protocol: A group with 3 Sham and 3 Nx rats was treated with 250 mg/kg/day of RP and, another group with 3 Sham and 3 Nx rats received 150 mg/kg/day of RP for 30 days by gavage. Additionally, 3 Sham and 3 Nx were kept untreated and used as controls. The group treated with the higher dose of propolis (250 mg/kg/day) presented lower body weight when compared to their respective control groups, while rats treated with 150 mg/kg/day of RP presented body weight values similar to those observed in their respective control groups. No rats died, and no changes in the regular behavior of animals were observed.

### Experimental Protocol

One month after renal ablation, tail-cuff pressure (TCP) and daily urinary protein excretion (Uprot, mg/24h) were determined for all animals. TCP was determined by an automated method (LE 5002, Panlab, Spain), after preconditioning (at least twice), under light restraining and after twenty minutes of warming in a silent room. TCP values were the average of at least five consecutive measurements obtained after stabilization of signal. At this time, Nx rats with TCP lower than 145 mmHg and Uprot lower than 50 mg/24h were excluded from the study. Remaining Nx animals were then divided in two groups (Untreated and RP) in such a way that the variation of Uprot and TCP did not exceed 6% between the pairs of groups. All animals were followed for two additional months (treatment period), with daily assessment of body weight (BW, g), and monthly assessment of Uprot and TCP. At the end of the study, rats were anesthetized with intraperitoneal injection of Ketamin 5% (50 mg/kg) and Xylazin 2% (0.5 ml/kg), in the ratio of 8:1, and blood samples were drawn from cardiac puncture for biochemical analysis. The left kidney was then retrogradely perfused in situ with Duboscq-Brazil solution, after a brief washout with saline to remove blood from renal tissue. After perfusion-fixation, the kidneys were weighted and two midcoronal slices of kidney were post-fixed in buffered 10% formaldehyde solution. Renal tissue was then embedded in paraffin by standard sequential techniques, for further assessment of glomerular and interstitial injury, as well as for immunohistochemical analysis.

### Histological and Immunohistochemical Analysis

For all histomorphometric and immunohistochemical analysis we employed 4-μm-thick renal sections; which were initially deparaffinized and rehydrated using standard techniques. To assess glomerular injury, sections were stained with periodic acid-Schiff (PAS) protocol. Glomerulosclerosis (GS) was defined as the presence of dense and abundant deposition of PAS-positive material in the glomerular tuft, leading to occlusion of capillary loops and segmental hyalinization. The GS extent was evaluated by two different methods: 1) Determination of the percentage of glomeruli exhibiting sclerotic lesions (%GS); 2) Calculation of a GS index (GSI) for each rat by attributing a score to each glomerulus and computing a weighted average of these scores, as described previously [14]. For the calculation of either %GS or the GSI, at least 120 consecutive glomeruli were examined for each rat. The fractional cortical interstitial area (INT) was evaluated in Masson-stained sections. Twenty-five consecutive pictures of microscopic fields (at x100 magnification) were evaluated and the renal cortical interstitial area positively stained by Masson was measured in pixels by image processing software (Image-Pro Plus 4.5). All histomorphometric evaluations were performed blindly by a single observer. For immunohistochemical detection of macrophages (ED-1^+^) and angiotensin II positive interstitial cells (AII^+^), sections underwent antigen retrieval, by steaming for twenty minutes in citrate buffer (pH = 6.0), followed by endogenous peroxidase and biotin blocking. In order to prevent nonspecific binding, sections were pre-incubated with 5% normal rabbit/horse serum before over night incubation with the following primary antibodies; monoclonal mouse anti-rat ED-1 antibody (Serotec, MCA341R), to detect monocytes and macrophages, and polyclonal rabbit anti-Angiotensin II (Peninsula, T-4007) to detect AII^+^ cells. Omitting incubation with the primary antibody was performed as negative control experiments. After washing, sections employed in the detection of ED-1^+^ cells were incubated with Dako EnVision+System-HRP Labelled Polymer Anti-mouse (K4001) and developed with DAB Chromogen System (Dako). Meanwhile, sections used to detect AII^+^ cells were incubated with a polyclonal biotinylated goat anti-rabbit antibody (Vector, BA1000) followed by incubation with the Vectastain ABC-AP kit (Vector, AK5000) and developed with Permanent Red Substrate-Chromogen (Dako, K0640). Finally, all slides were counterstained with Mayer’s hemalaum, and covered with Kaiser’s glycerin–gelatin (Merck, Darmstadt, Germany). Cortical interstitial macrophages, as well as angiotensin II positive cells were evaluated by counting the number of ED-1^+^ or AII^+^ cells per microscopic field. Twenty-five microscopic fields at x200 magnifications were examined for each section. Additionally, the extent of macrophage infiltration in glomerulus was evaluated by counting the number of ED-1 positive cells/glomerulus, in at least, 25 consecutive glomeruli, also observed under x200 magnification, for each section.

### Biochemical analysis and reactive oxygen metabolites production

Serum creatinine (Cr) and potassium (K^+^) concentrations were evaluated in arterial blood samples using commercially available kits. Urinary levels of Tbars were determined using a thiobarbituric acid assay. In brief, a 0.2-mL urine sample was diluted in 0.8 mL of distilled water. Immediately thereafter, 1 mL of 17.5% trichloroacetic acid was added. All samples were kept on ice during this stage. Subsequently, 1 mL of 0.6% thiobarbituric acid (pH = 2) was added, and the sample was placed in a boiling water bath for 15 min, after which it was allowed to cool. We then added 1 mL of 70% trichloroacetic acid and incubated the mixture for 20 min. The sample was then centrifuged for 15 min at 2,000 rpm. The optical density of the supernatant was read at 534 nm against a blank reagent using a spectrophotometer. The quantity of Tbars was calculated using a molar extinction coefficient of 1.56 × 10^5^ mol^−1^/cm^−1^ and expressed as nmol/24h.

### Statistical Analysis

Data was expressed as mean ± standard deviation (SD). Differences among groups were analyzed using one-way ANOVA with Newman-Keuls posttest using Graph-Pad Prism (version 5.0). Statistical significance was established at p < 0.05.

## Results

In accordance to previous results, thirty days after renal ablation, Nx rats exhibited lower body weight when compared to S animals. Moreover, Nx rats presented hypertension and increased proteinuria, and all these differences were statistically significant as shown in Table 1.

**Table 1:** Renal and functional parameters observed 30 days after renal ablation, immediately before starting of treatments (pretreatment).

	**N**	**BW (g)**	**TCP (mmHg)**	**Uprot (mg/24h)**
**S**	08	293 ± 33	118 ± 8	16.7 ± 6.4
**S + RP**	08	291 ± 20	114 ± 3	17.1 ± 6.2
**Nx**	11	265 ± 27^ab^	156 ± 11^ab^	83.7 ± 37.4^ab^
**Nx + RP**	08	246 ± 31^ab^	168 ± 16^ab^	99.5 ± 32.9^ab^

Values are presented as mean ± SD. The Number of animals used in each group (N), body weight (BW), tail cuff pressure (TCP), urinary protein excretion (Uprot).

**^a^**p < 0.05 vs. group S

**^b^**p < 0.05 vs. group S+RP

**^c^**p < 0.05 vs. group Nx.

**Figure 1:**
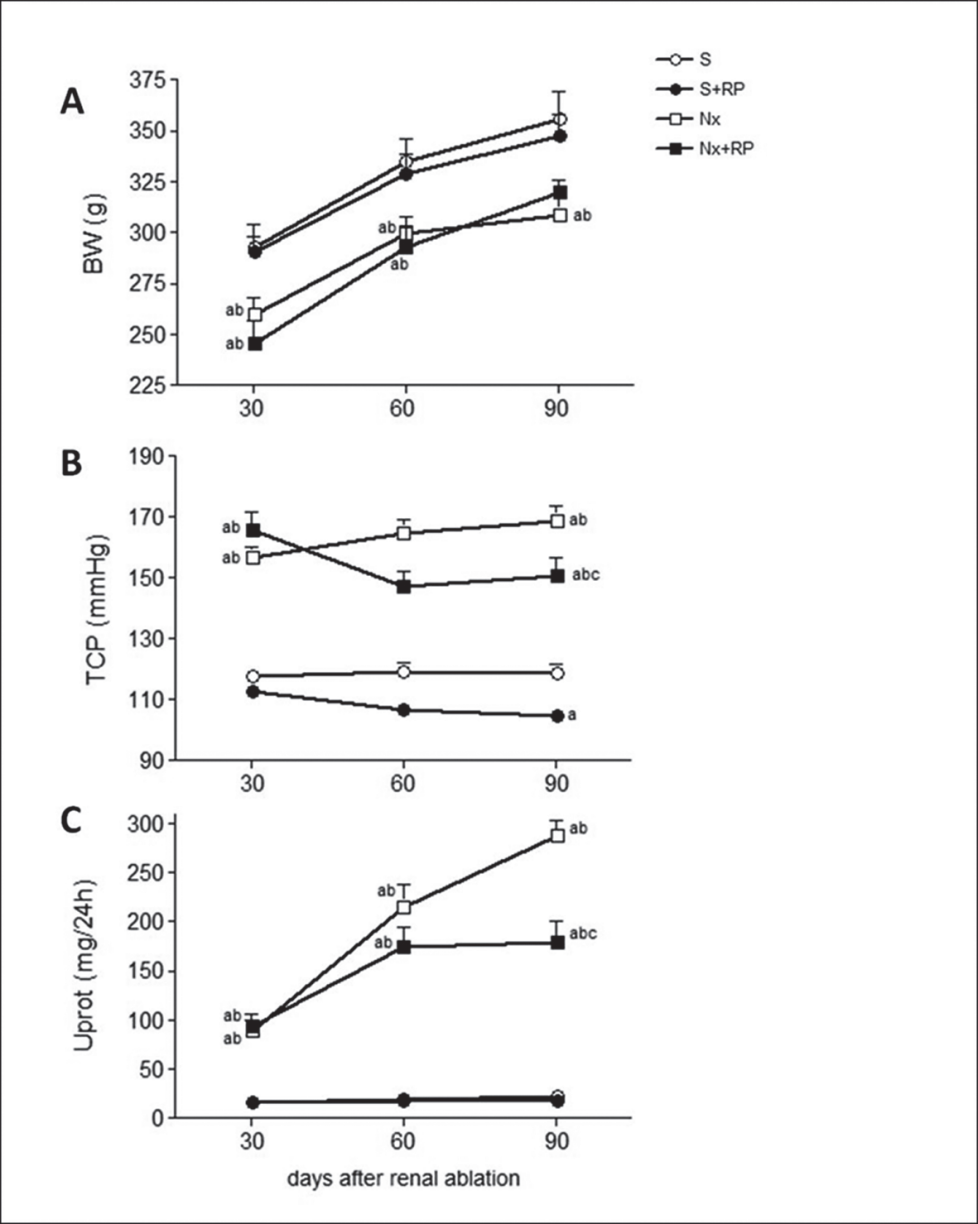
Time course of body weight (A), tail-cuff pressure (B) and proteinuria (C). S (open circles), S+RP (filled circles), Nx (open squares) and Nx+RP (filled squares). Comparisons were performed only between groups in the same analysis times. ^**a**^p < 0.05 vs. group S, ^**b**^p < 0.05 vs. group S+RP, ^**c**^p < 0.05 vs. group Nx.

There was no mortality in S groups until the end of the study. However, in the untreated Nx group, the survival rate was only 41%. As can be verified in Table 2, Nx+RP group demonstrated a 26% higher survival rate compared to untreated Nx animals. During all the experimental period, Nx rats presented reduced body growth compared to S animals, and the RP treatment did not changed this parameter (Fig. 1A). Hypertension, as well as proteinuria exhibited by Nx rats 30 days after renal ablation, did worse in untreated animals by the end of the study (90 days after surgery), as can be seen in Table 2 and Fig. 1. RP treatment significantly reduced TCP in both S and Nx animals, and partially prevented the progression of proteinuria in Nx+RP rats, after 60 days of administration. Heart weight was analyzed at the end of the study, after the procedure of perfusion-fixation. In accordance with the behavior of systemic blood pressure, Nx rats exhibited increased heart weight / body weight ratio when compared to S groups. RP treatment partially prevented the heart hypertrophy in the Nx+RP group; however, this effect was not statistically significant (Table 2).

**Table 2:** Renal and functional parameters after 60 days of RP treatment (90 days after renal ablation).

	**Survival (%)**	**BW (g)**	**TCP (mmHg)**	**Uprot (mg/24h)**	**HW/BW∙10^3^**	**Cr (mg/24h)**	**Tbars (nmol/24h)**
**S**	100	357 ± 38	118 ± 2	22.1 ± 7.6	3.47 ± 0.33	0.33 ± 0.15	48 ± 23
**S + RP**	100	349 ± 29	106 ± 5^a^	18.1 ± 3.3	3.63 ± 0.41	0.28 ± 0.05	47 ± 28
**Nx**	40.7^a^	309 ± 33^ab^	169 ± 16^ab^	287.9 ± 49.6^ab^	5.20 ± 0.86^ab^	2.15 ± 1.78^ab^	421 ± 297^ab^
**Nx + RP**	66.7^ab^	320 ± 17	151 ± 16^abc^	179.3 ± 60.8^abc^	4.58 ± 0.9^ab^	1.0 ± 0.5^c^	275± 120^abc^

Values are presented as mean ± SD. Percentage of Survival (%), body weight (BW), tail cuff pressure (TCP), urinary protein excretion (Uprot), heart weight / body weight ratio (HW/BW∙10^3^), serum creatinine concentration (Cr), urinary levels of reactive oxygen metabolites (Tbars).

**^a^**p < 0.05 vs. group S

**^b^**p < 0.05 vs. group S+RP

**^c^**p < 0.05 vs. group Nx.

**Figure 2:**
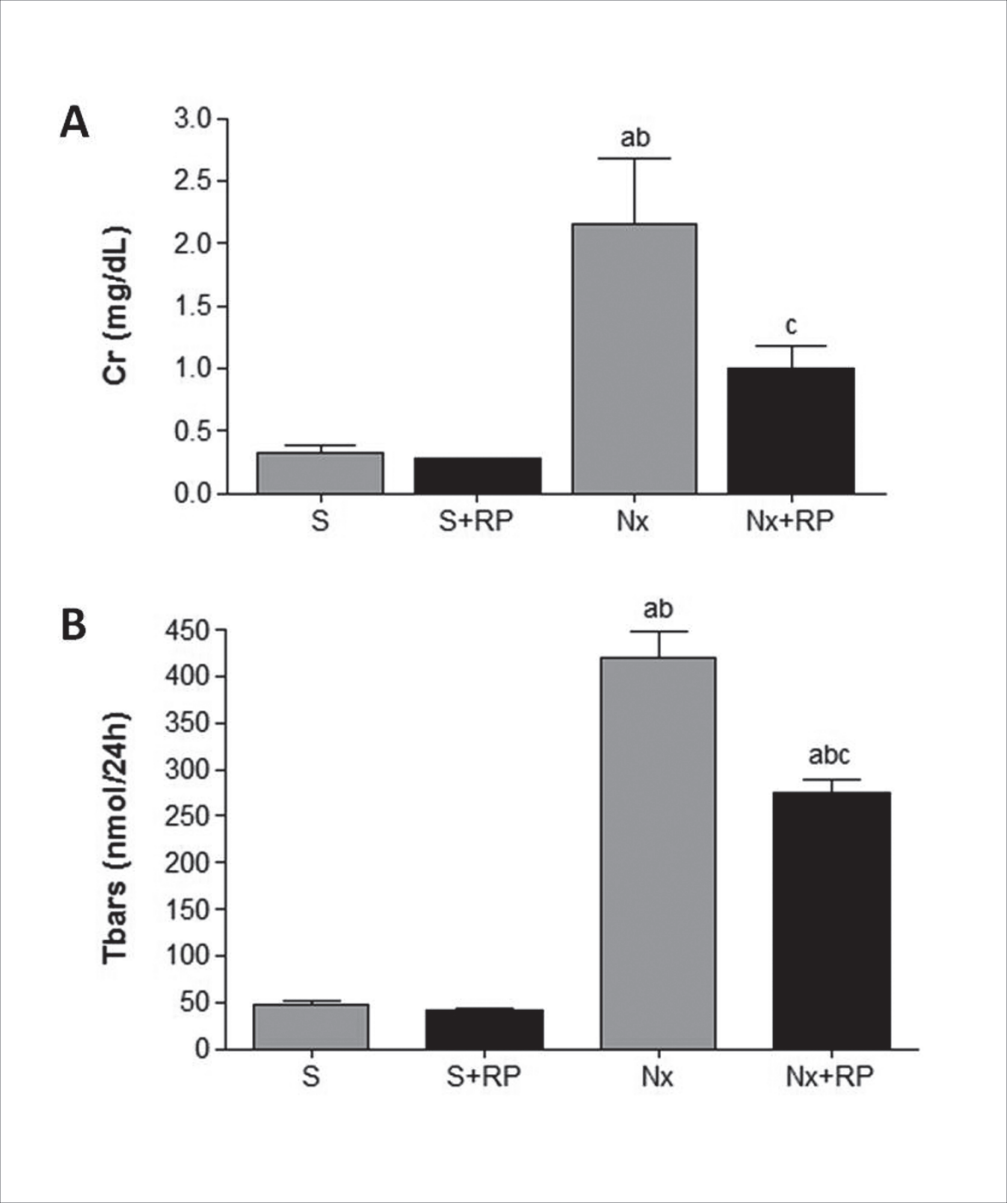
Renal function and reactive oxygen metabolites after 60 days of RP treatment (90 days after renal ablation). Bar graphs of serum creatinine concentration (A) and urinary levels of reactive oxygen metabolites Tbars (B). S (open circles), S+RP (filled circles), Nx (open squares) and Nx+RP (filled squares). ^**a**^p < 0.05 vs. group S, ^**b**^p < 0.05 vs. group S+RP, ^**c**^p < 0.05 vs. group Nx.

**Figure 3:**
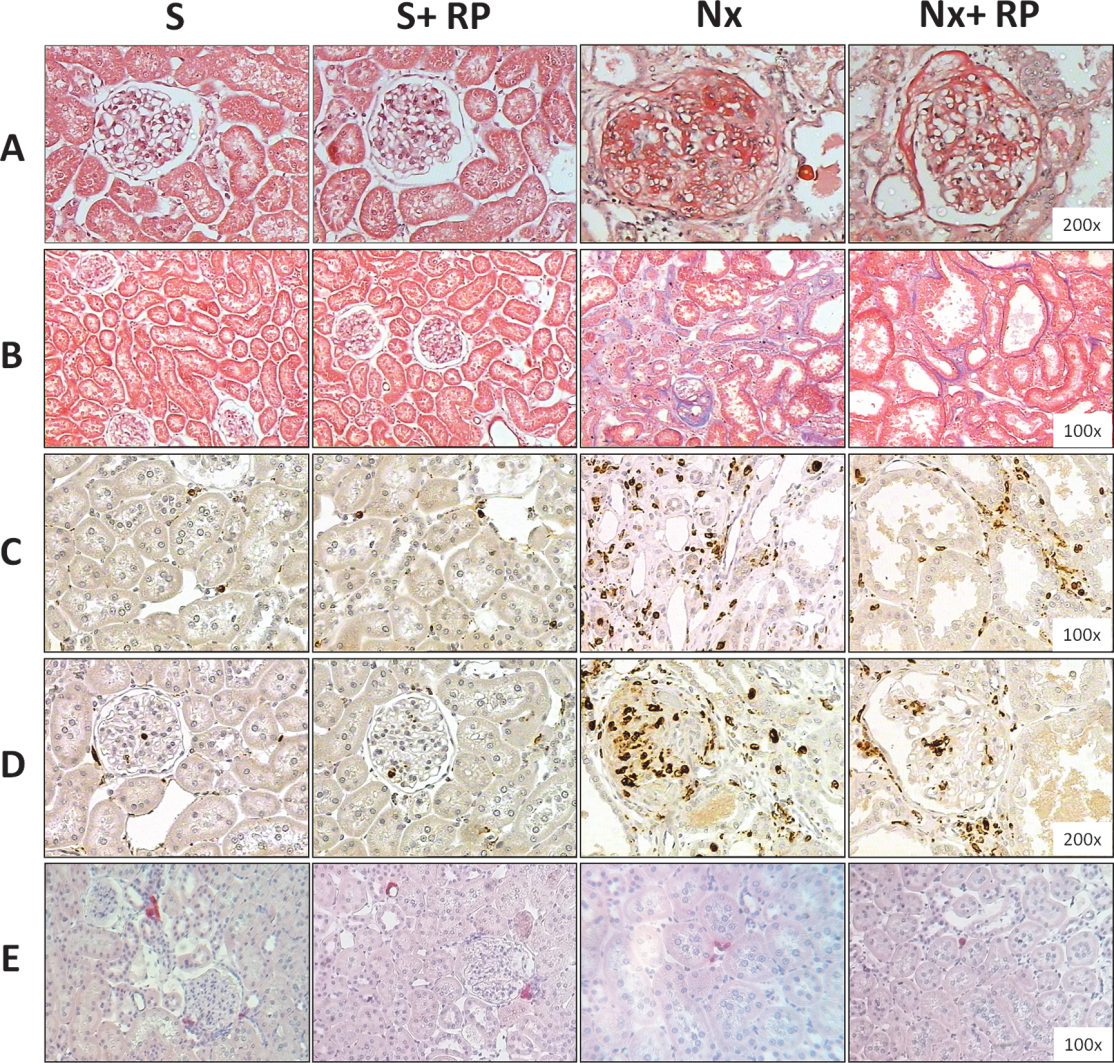
Representative panel of histological and immunohistochemical analysis. Microphotographies of: PAS staining, to assessment of glomerular lesions (A), Trichromic Masson staining, to evaluate interstitial expansion (B), ED-1 immunohistochemistry, to analyze macrophage infiltration in the interstitial area (C) and in the glomeruli (D), and AII immunohistochemistry, to evaluate the presence of renal cortical interstitial cells positive to AII (E).

**Figure 4:**
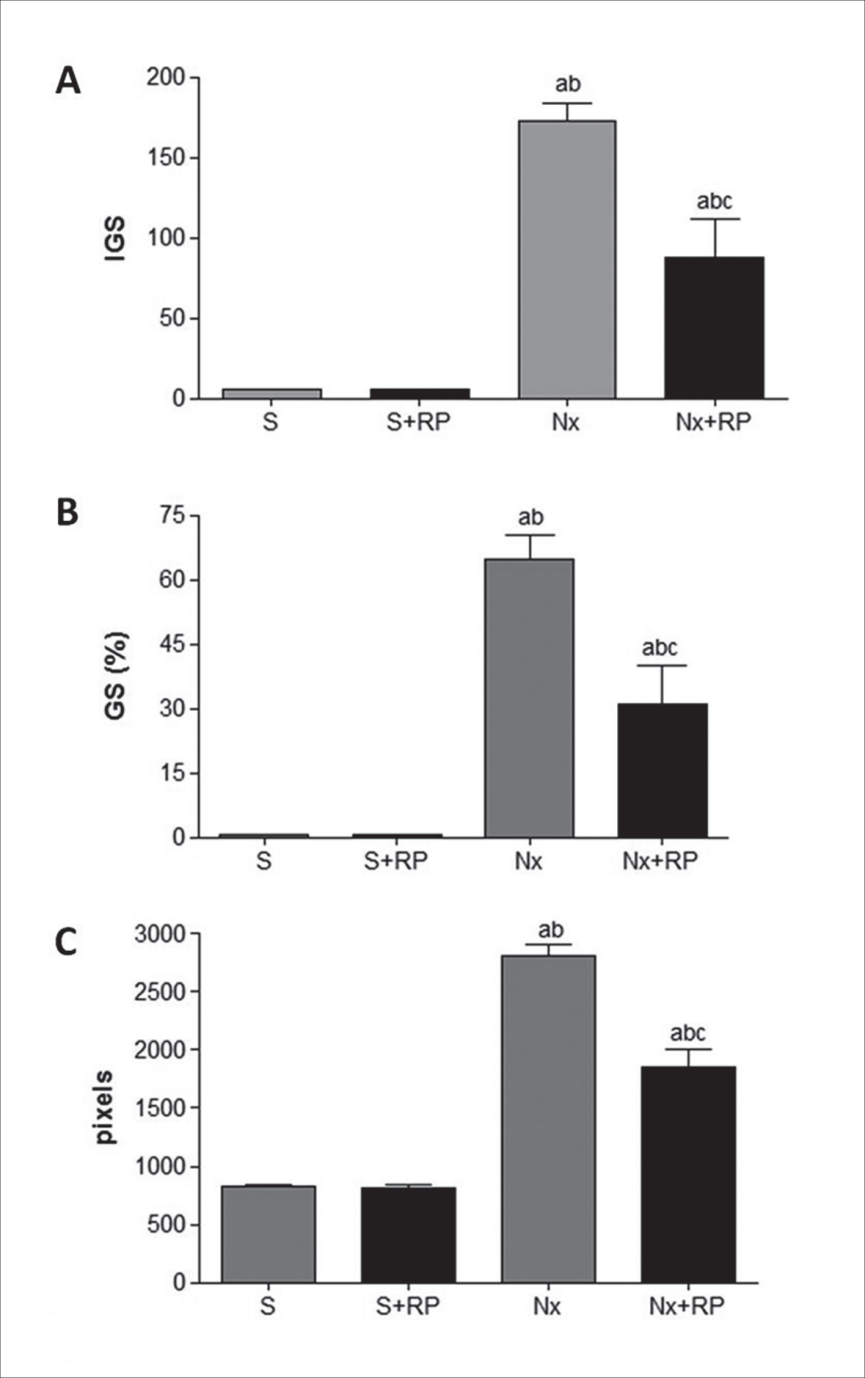
Histological parameters after 60 days of RP treatment (90 days after renal ablation). Bar graphs of glomerulosclerosis index (A), percentage of sclerotic glomeruli (B) and interstitial expansion (C). ^**a**^p < 0.05 vs. group S, ^**b**^p < 0.05 vs. group S+RP, ^**c**^p < 0.05 vs. group Nx.

One of the main features of renal ablation model is the glomerular structural and functional deterioration. Accordingly, Nx untreated rats presented elevated serum creatinine concentration, as well as increased percentage of sclerotic glomeruli and high index of glomerulosclerosis, as represented in tables 2 and 3 and Figs. 2A, 3A and 4A/B. Both renal function loss and structural glomerular damage were significantly attenuated by RP treatment. Moreover, serum creatinine concentration levels of Nx+RP group did not differ statistically from those presented by S groups.

**Table 3:** Histological parameters after 60 days of RP treatment (90 days after renal ablation).

	**GS (%)**	**IG (index)**	**INT (pixels)**	**ED-1 (^+^cells/field)**	**ED-1 (^+^cells/glomerulus)**	**AII (^+^cells/field)**
**S**	0.45 ± 0.3	6.1 ± 0.1	820 ± 43	0.67 ± 1.4	0.28 ± 0.1	0.14 ± 0.08
**S + RP**	0.45 ± 0.3	6.1 ± 0.1	815 ± 58	0.60 ± 1.0	0.38 ± 0.1	0.04 ± 0.02
**Nx**	64.8 ± 17.9^ab^	173 ± 34^ab^	2808 ± 330^ab^	8.14 ± 3.3^ab^	3.28 ± 1.7^ab^	0.43 ± 0.16^b^
**Nx + RP**	31.1 ± 25.1^abc^	87.3 ± 69.7^abc^	1853 ± 416^abc^	5.18 ± 2.1^abc^	1.28 ± 0.5^abc^	0.19 ± 0.08

Values are presented as mean ± SD. Percentage of Sclerotic glomeruli (GS%), Glomerulosclerosis Index (IG) Masson positive cortical interstitial area (INT), interstitial and glomerular macrophage infiltration (ED-1) and interstitial cells positive to angiotensin II (AII).

**^a^**p < 0.05 vs. group S

**^b^**p < 0.05 vs. group S+RP

**^c^**p < 0.05 vs. group Nx.

**Figure 5:**
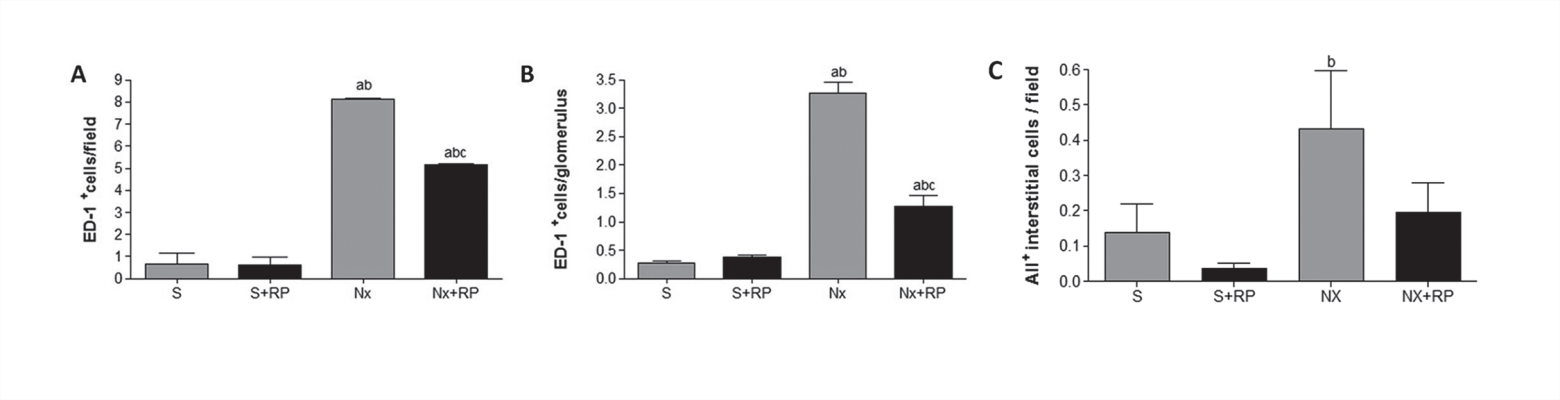
Immunohistochemical analysis after 60 days of RP treatment (90 days after renal ablation). Bar graphs of interstitial (A) and glomerular (B) macrophage infiltration, and AII positive interstitial cells (C) ^**a**^p < 0.05 vs. group S, ^**b**^p < 0.05 vs. group S+RP, ^**c**^p < 0.05 vs. group Nx.

Renal interstitial inflammation and fibrosis are some of the histopathological features commonly observed in the Nx model. In the present study, we evaluate cortical interstitial expansion as positive Masson staining area, which is demonstrated in table 3 and illustrated in Fig. 3B. The quantification of this parameter (Fig. 4C) showed increased interstitial area in untreated Nx group. RP treatment partially prevented the progression of renal fibrosis, which was significantly lower in Nx+RP rats compared to Nx animals. In order to investigate interstitial and glomerular inflammation we performed immunohistochemical analysis for ED-1 and AII positive cells (Table 3 and Fig. 3). Both interstitial and glomerular renal compartments presented high macrophage infiltration in untreated Nx animals when compared to S group. Once more, RP treatment partially prevented renal inflammation. Nx+RP group showed significantly less macrophage infiltration than untreated Nx rats. Additionally, immunohistochemical analysis detected also the presence of AII^+^ cells in the renal interstitium of NX animals, however, RP treatment did not significantly changed this parameter (Fig. 5).

We verified a possible antioxidant response of Nx animals to RP treatment by measuring the urinary concentration of reactive oxygen metabolites (Tbars) in all groups by the end of the study. As shown in Fig. 2, both untreated and RP treated rats exhibited elevated urinary levels of Tbars. However, RP treatment promoted a significant reduction of Tbars excretion in Nx+RP rats when compared to untreated group. All individual results obtained in this study and necessary to replicate our statistical analysis were provided as S1 Table.

## Discussion

Corroborating previous studies after 30 days of renal ablation, Nx animals exhibited hypertension and elevated proteinuria, suggesting that RP treatment was initiated when Nx animals already presented a well established CKD process [5,6,18]. We demonstrated that RP treatment reduced blood pressure levels, partially prevented the progression of proteinuria and attenuate renal function loss and histological damage in the remnant kidney. These findings were accompanied by reduction of renal cortical macrophage infiltration and decrease of urinary Tbars excretion.

We believe that studies employing propolis as a potential treatment in nephropathies have been discouraged due to the description of sporadic reports of acute nephrotoxicity caused by this drug, probably mediated by immunoallergic mechanisms [19]. Moreover, it is well known that some of the most employed drugs in the battle against CKD progression (ACE inhibitors, AT1R blockers) can, controversially, cause acute kidney injury due to interstitial nephritis or hemodynamic mechanisms in some situations [20]. Propolis was already shown to present antihypertensive effects in previous studies employing spontaneously hypertensive rats (SHR) [14–16,21]. This natural compound also exhibited hypoglycemiant properties and other metabolic effects in experimental models of diabetes [22–24]. However, no one of these mentioned studies explored the potential renoprotective effects of propolis. To the best of our knowledge this is the first study to address the possible protective effect of RP in a CKD model. Biological properties of propolis are still unclear and may depend on its chemical composition, which varies especially regarding to the polyphenols, according to the region where it is produced [13]. This diversity depends on local factors such as vegetation and the types of bees that produce it [25,26]. Recently, Silva et al characterized by HPLC the main constituents of RP we used in our study. In this analysis; medicarpin isoflavones and 3-Hydroxy-8, 9-dimethoxypterocarpan were mainly found, the latter, accounting for 60% of the constitution of the compound [17]. Unfortunately, the individual activity of RP components was not evaluated. Therefore, it was not clarified if one of them could achieve the same effect if tested alone.

There is abundant evidence that blood pressure control is one of the most effective strategies to prevent the progression of chronic nephropathies [27–29]. Lower blood pressure leads to less proteinuria and less glomerular damage in both humans and experimental models of CKD [27–31]. Effectively, the significant decrease of systemic blood pressure promoted by RP treatment may have contributed to the reduction of proteinuria and renal damage observed in Nx+RP animals. The mechanism by which propolis reduces blood pressure is still unclear. Kubota et al, using aorta of SHR suggest that the hypotensive effect caused by propolis may be mediated by acetylcholine vasodilation action [16]. Whereas, Mishima et al demonstrated a significant reduction in blood pressure of SHR when treated with propolis extracts rich in caffeoylquinic acids [21]. In another study using isolated rat aorta, Cicala et al demonstrated that the addition of caffeic acid, a major component of propolis found in Asia and Europe, inhibited the vasoconstrictor response to phenylephrine and potassium chloride [32]. Propolis flavonoids derivatives as dihydrokaempferide, betuletol and especially isosakuranetin, also demonstrate antihypertensive effect in SHR [14,15]. However, it is noteworthy that in SHR, the most widely used model of essential hypertension, there is scarce glomerular and interstitial lesion compared to remnant kidney [14,15]. The Nx model, on the other hand, is characterized by significant renal damage, with resistant hypertension, rising proteinuria, severe glomerulosclerosis, interstitial fibrosis and progressive renal function loss [7,8,10,18]. The sustained reduction in blood pressure levels of Nx+RP animals reinforce the significant antihypertensive effect of propolis. Although it remains unclear whether the lowered blood pressure was a cause or a consequence of renoprotection, we also observed a significant reduction of this parameter in S animals treated with RP (control group). This suggests that the antihypertensive mechanism of propolis was not exclusively dependent on the preservation of renal function.

The role of inflammatory mechanisms in the pathogenesis of chronic kidney disease and hypertension has been strongly suggested [5–8]. The presence of lymphocyte and macrophage infiltration in the renal tissue is a constant finding in a variety of nephropathies [18,27,34,36]. As expected, we observed a significant increase in the expression of macrophages in both interstitial and glomerular renal compartments in the Nx group. RP treatment reduced significantly this parameter. Previous evidence demonstrated that treatment of Nx rats with the anti-inflammatory nitroflurbiprofen reduces urinary albumin excretion, macrophage infiltration and glomerulosclerosis without any effect on blood pressure [6]. In turn, Utimura et al reported that the use of mycophenolate mofetil, an anti-inflammatory drug, reduced proteinuria and glomerular sclerosis in experimental diabetic nephropathy, with no changes in systemic blood pressure [7].

Oxidative stress is also considered an important pathway in the progression of CKD [9,30,31,33,35]. It has already been shown that the use of antioxidant drugs can exert renoprotective effects in experimental studies [37–39]. The antioxidant potential of propolis has been amply demonstrated [25–27,40,41]. Tohamy et al, using a model of cisplatin nephrotoxicity, in which the production of catalase and glutathione in the renal tissue is significantly reduced, demonstrated that propolis treatment reestablished the production of these endogenous antioxidant enzymes [42]. Additionally, it was recently shown that propolis administration reduced plasma malondialdehyde production in the renal tissue of streptozotocin-induced diabetic rats [43]. We demonstrated a significant reduction in urinary Tbars excretion, a biochemical lipid peroxidation marker, in Nx animals treated with RP. This finding strongly suggests that RP extracts exhibit potent antioxidant properties and it may have contributed to anti-inflammatory response achieved in this CKD model.

Previous observations also suggest the involvement of oxidative stress in the pathogenesis of hypertension [35,44–46]. One of the proposed mechanisms is the interaction between oxidative stress and the renin angiotensin aldosterone system (RAAS). According to this theory, mesangial cells stimulated with angiotensin II (AII) could produce superoxide anions [45]. In this context, the use of inhibitors of the RAAS could reduce the production of reactive oxygen species (ROS) [47,48]. In this direction, Banday and Lokhandwala demonstrated that mice treated with an oxidizing agent, develop hypertension and increased expression of AT-1 receptors in the proximal tubules. These authors also demonstrated that the use of the oxidizing agent sensitizes the cells of proximal tubules to the effects of AII, particularly increased expression of sodium transporters [49]. In our study, RP treatment promoted a slight reduction in the number of renal cortical interstitial cells positive to AII, in both S and Nx animals. However, this results was not statistically significant.

It is hard to determine whether RP promoted a reduction in blood pressure and this hemodynamic effect was the responsible for reduced glomerular damage and interstitial inflammation in the renal parenchyma, or if the anti inflammatory and antioxidant effects of RP limited the hemodynamic changes in Nx model. As extensively demonstrated by Zatz R and Fujihara CK, glomerular hypertension is one of the most deleterious injury to kidneys in a variety of experimental models of CKD such as diabetic nephropathy, and the Nx model [6,7]. There are abundant evidences that blood pressure control is one of the most effective strategies to prevent the progression of chronic nephropathies. Lower blood pressure leads to less proteinuria and less glomerular damage in both humans and experimental models of CKD. Effectively, the significant decrease of systemic blood pressure promoted by RP treatment may have contributed to the reduction of proteinuria and renal damage observed in Nx+RP animals. However, due to some limitations of our study such as the lack of glomerular capillary blood pressure (PGC) analysis and the absence of a pressoric control Nx group (Nx rats treated only with antihypertensive drugs to achieve systolic blood pressure values similar to those obtained with RP treatment) it is not possible to evaluate the isolated influence of blood pressure control in the renoprotection observed in NX+RP animals. To clarify the specific mechanisms of RP in the development and evolution of CKD, further sectional studies, in which temporal analysis of both hemodynamic and inflammatory factors could be carried out, are strongly required.

It is important to observe that renal protection achieved with RP treatment in Nx rats was only partial, since the levels of proteinuria stabilized, but did not reduce to normal levels, as well as creatinine retention and other histological parameters. However, as exposed before, the 5/6 renal ablation can be considered a severe CKD model, and even treatments with drugs already established in combating hypertension and proteinuria as Losartan or Enalapril also fail to completely inhibit the progression of CKD in Nx rats when in monotherapy. [18,28].

## Conclusion

In conclusion, the use of Red Propolis reduced, at least partially, hypertension, proteinuria and serum creatinine retention in the Nx animals, as well as the glomerular damage and interstitial expansion. These findings were accompanied by a reduction in macrophage infiltration and oxidative stress. It was the first time that RP renoprotective effects have been demonstrated in a severe CKD model. This renoprotection might be related to the reduction of renal inflammation and oxidative stress. However, additional studies are required to completely clarify the mechanisms by which RP exerts its benefic effects.

## Supporting Information

S1 Table Individual results obtained from each rat included in the study. Values represent the individual measures obtained from each animal in each evaluated parameter. The acronyms of experimental groups (S, S+RP, NX, NX+RP) are in red, in the top of the second column. The numbers in bold below each acronym are the rats identification. SD: standard deviation, SE: standard error and N: number of animals analyzed regarding each parameter.
(XLS)
